# 1-Benzyl­piperazine-1,4-diium bis­(perchlorate) monohydrate

**DOI:** 10.1107/S1600536810023123

**Published:** 2010-06-23

**Authors:** Kamel Kaabi, Meher El Glaoui, Erwann Jeanneau, Mohamed Rzaigui, Cherif Ben Nasr

**Affiliations:** a Laboratoire de Chimie des Matériaux, Faculté des Sciences de Bizerte, 7021 Zarzouna, Tunisia; b Université Lyon1, Centre de Diffractométrie Henri Longchambon, 43 Boulevard du 11 Novembre 1918, 69622 Villeurbanne Cedex, France

## Abstract

In the title compound, C_11_H_18_N_2_^2+^·2ClO_4_^−^·H_2_O, one perchlor­ate anion is disordered over two orientations in a 0.66 (3):0.34 (3) ratio. Inter­molecular O—H⋯O, N—H⋯O and C—H⋯O hydrogen bonds link the cations, anions and water mol­ecules into ribbons extending along [100].

## Related literature

For general background to the properties of perchlorate salts containing organic cations, see: Czarnecki *et al.* (1994 ▶); Czupinski *et al.* (2002 ▶, 2006 ▶). For related structures, see: Antolini *et al.* (1982 ▶); Place & Willett (1988 ▶).
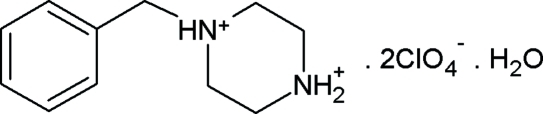


## Experimental

### 

#### Crystal data

C_11_H_18_N_2_^2+^·2ClO_4_^−^·H_2_O
*M* *_r_* = 395.19
Triclinic, 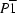

*a* = 8.6632 (6) Å
*b* = 10.0197 (8) Å
*c* = 10.8831 (7) Å
α = 70.184 (7)°
β = 83.946 (6)°
γ = 70.560 (7)°
*V* = 838.05 (12) Å^3^
*Z* = 2
Mo *K*α radiation
μ = 0.44 mm^−1^
*T* = 293 K
0.53 × 0.40 × 0.25 mm

#### Data collection

Oxford Diffraction Xcalibur Atlas Gemini ultra diffractometer
Absorption correction: analytical (*CrysAlis PRO*; Oxford Diffraction, 2006 ▶) *T*_min_ = 0.832, *T*_max_ = 0.907
32031 measured reflections
5885 independent reflections
3882 reflections with *I* > 2σ(*I*)
*R*_int_ = 0.045

#### Refinement

*R*[*F* ^2^ > 2σ(*F* ^2^)] = 0.066
*wR*(*F* ^2^) = 0.240
*S* = 1.12
5885 reflections
255 parameters
40 restraints
H-atom parameters constrained
Δρ_max_ = 1.04 e Å^−3^
Δρ_min_ = −0.88 e Å^−3^

### 

Data collection: *CrysAlis PRO* (Oxford Diffraction, 2006 ▶); cell refinement: *CrysAlis PRO*; data reduction: *CrysAlis PRO*; program(s) used to solve structure: *SIR97* (Altomare *et al.*, 1999 ▶); program(s) used to refine structure: *SHELXL97* (Sheldrick, 2008 ▶); molecular graphics: *CAMERON* (Watkin *et al.*, 1996 ▶); software used to prepare material for publication: *SHELXL97*.

## Supplementary Material

Crystal structure: contains datablocks global, I. DOI: 10.1107/S1600536810023123/cv2717sup1.cif

Structure factors: contains datablocks I. DOI: 10.1107/S1600536810023123/cv2717Isup2.hkl

Additional supplementary materials:  crystallographic information; 3D view; checkCIF report

## Figures and Tables

**Table 1:** Hydrogen-bond geometry (Å, °)

*D*—H⋯*A*	*D*—H	H⋯*A*	*D*⋯*A*	*D*—H⋯*A*
O9—H91⋯O4*A*^i^	0.81	2.27	2.942 (11)	141
O9—H92⋯O5^ii^	0.82	2.06	2.869 (6)	170
N16—H161⋯O8	0.90	2.15	2.964 (3)	151
N19—H191⋯O9^iii^	0.89	1.92	2.750 (4)	155
N19—H192⋯O1*A*^iv^	0.89	2.08	2.907 (10)	154
C17—H172⋯O6^v^	0.97	2.48	3.446 (5)	172
C20—H201⋯O7^iv^	0.95	2.49	3.406 (4)	160
C21—H212⋯O3*A*	0.96	2.48	3.130 (15)	125

Symmetry codes: (i) 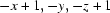; (ii) 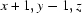; (iii) 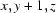; (iv) 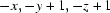; (v) 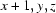.

**Fig. 1.**
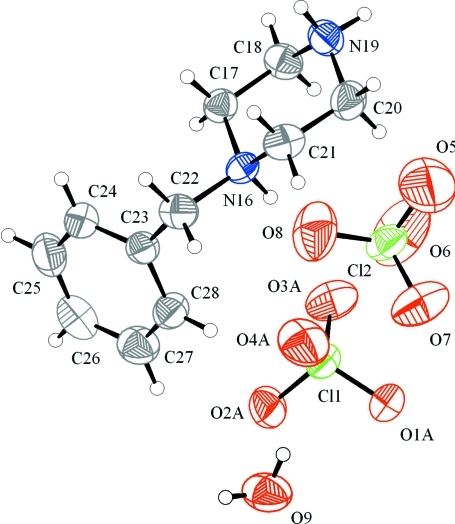
View of (I), showing 50% probability displacement ellipsoids and arbitrary spheres for the H atoms. For the disordered perchlorate anion, only major part is shown.

## supplementary crystallographic information

### Comment

Chemists and physicists of the solid state have shown an increasing interest in the study of perchlorate salts containing organic cations in recent years owing to their great interesting properties such as ferroelectric and dielectric behaviours. (Czarnecki *et al.*, 1994; Czupinski *et al.*, 2002; Czupinski *et al.*, 2006). Here, we report the synthesis and the crystal structure of the title compound (I), [C_11_H_18_N_2_]^2+^.2ClO_4_^-^.H_2_O.

The crystal structure of (I) (Fig.1), contains two ClO_4_^-^ anions, a 1-benzylpiperazine-1,4-diium dication and a water molecule. In its atomic arrangement, the ClO_4_^-^ anions are associated per pair *via* O—H···O hydrogen bonds generated by a water molecule to form [Cl_2_O_8_H_2_O]^2-^ entities. The 1-benzylpiperazine-1,4-diium dications are associated to these entities and connected them through N—H···O and C—H···O hydrogen bonds, leading to the formation of three dimensional network. As expected, the ClO_4_ anion has typical tetrahedral geometry where the Cl—O bond lengths and O—Cl—O angles are not equal to one another but very with the environment around the O atoms. In the title compound, the Cl—O bond lengths vary from 1.382 (12) Å to 1.437 (7) Å for Cl1O_4_ anion and from 1.374 (3) Å to 1.484 (4) Å for Cl2O_4_anion. The O—Cl—O angles range from 104.2 (14) ° to 119.3 (15) ° for the first anion and from 103.1 (2) °. to 118.4 (2) ° for the second one. These values clearly indicate that the coordination geometry of the Cl atom can be regarded as being a distorted tetrahedron. However, for Cl2O_4_ tetrahedron all the oxygen atoms are involved in hydrogen bonds, while only three oxygen atoms acts as acceptors of hydrogen bonds for the Cl1O_4_ tetrahedron.

### Refinement

All H atoms were located in a difference map, but those attached to carbon atoms were repositioned geometrically. The H atoms were initially refined with soft restraints on the bond lengths and angles to regularize their geometry (C—H in the range 0.93–0.98, N—H in the range 0.86–0.89 N—H to 0.86 O—H = 0.82 Å) and *U*_iso_(H) (in the range 1.2–1.5 times *U*_eq_ of the parent atom), after which the positions were refined with riding constraints. The rotational disorder observed for one perchlorate anion (with Cl1) was modelized using two superimposed molecules with partial occupancies. The molecules were then refined with restraints on the Cl—O bonds, O—Cl—O angles and displacement parameters of the oxygen atoms

### Crystal data

**Table:**

C_11_H_18_N_2_^2+^·2ClO_4_^−^·H_2_O	*Z* = 2
*M**_r_* = 395.19	*F*(000) = 412.000
Triclinic, *P*1	*D*_x_ = 1.566 Mg m^−^^3^
Hall symbol: -P 1	Mo *K*α radiation, λ = 0.7107 Å
*a* = 8.6632 (6) Å	Cell parameters from 13879 reflections
*b* = 10.0197 (8) Å	θ = 3.5–32.9°
*c* = 10.8831 (7) Å	µ = 0.44 mm^−^^1^
α = 70.184 (7)°	*T* = 293 K
β = 83.946 (6)°	Plate, colourless
γ = 70.560 (7)°	0.53 × 0.40 × 0.25 mm
*V* = 838.05 (12) Å^3^	

### Data collection

**Table:**

Oxford Diffraction Xcalibur Atlas Gemini ultra diffractometer	5885 independent reflections
Radiation source: Enhance (Mo) X-ray Source	3882 reflections with *I* > 2σ(*I*)
graphite	*R*_int_ = 0.045
Detector resolution: 10.4685 pixels mm^-1^	θ_max_ = 33.0°, θ_min_ = 3.5°
ω/2\ scans	*h* = −13→13
Absorption correction: analytical (*CrysAlis PRO*; Oxford Diffraction, 2006)	*k* = −15→15
*T*_min_ = 0.832, *T*_max_ = 0.907	*l* = −15→16
32031 measured reflections	

### Refinement

**Table:**

Refinement on *F*^2^	Secondary atom site location: difference Fourier map
Least-squares matrix: full	Hydrogen site location: inferred from neighbouring sites
*R*[*F*^2^ > 2σ(*F*^2^)] = 0.066	H-atom parameters constrained
*wR*(*F*^2^) = 0.240	*w* = 1/[σ^2^(*F*_o_^2^) + (0.15*P*)^2^ + 0.05*P*] where *P* = (*F*_o_^2^ + 2*F*_c_^2^)/3
*S* = 1.12	(Δ/σ)_max_ < 0.001
5885 reflections	Δρ_max_ = 1.04 e Å^−^^3^
255 parameters	Δρ_min_ = −0.88 e Å^−^^3^
40 restraints	Extinction correction: *SHELXL97* (Sheldrick, 2008), Fc^*^=kFc[1+0.001xFc^2^λ^3^/sin(2θ)]^-1/4^
Primary atom site location: structure-invariant direct methods	Extinction coefficient: 0.062 (11)

### Special details

**Table:**

Geometry. All e.s.d.'s (except the e.s.d. in the dihedral angle between two l.s. planes) are estimated using the full covariance matrix. The cell e.s.d.'s are taken into account individually in the estimation of e.s.d.'s in distances, angles and torsion angles; correlations between e.s.d.'s in cell parameters are only used when they are defined by crystal symmetry. An approximate (isotropic) treatment of cell e.s.d.'s is used for estimating e.s.d.'s involving l.s. planes.

### Fractional atomic coordinates and isotropic or equivalent isotropic displacement parameters (Å^2^)

**Table:**

	*x*	*y*	*z*	*U*_iso_*/*U*_eq_	Occ. (<1)
N16	0.2509 (2)	0.3896 (2)	0.78548 (16)	0.0377 (4)	
H161	0.1556	0.3721	0.7879	0.045*	
N19	0.1505 (3)	0.7088 (2)	0.6477 (2)	0.0507 (5)	
H191	0.2431	0.7311	0.6393	0.061*	
H192	0.0729	0.7939	0.6086	0.061*	
C17	0.2348 (3)	0.5002 (3)	0.8544 (2)	0.0419 (4)	
H171	0.2003	0.4603	0.9431	0.050*	
H172	0.3416	0.5114	0.8562	0.050*	
C18	0.1106 (3)	0.6496 (3)	0.7878 (2)	0.0490 (5)	
H181	0.1085	0.7216	0.8271	0.059*	
H182	0.0026	0.6393	0.7937	0.059*	
C20	0.1645 (4)	0.5982 (3)	0.5795 (2)	0.0560 (6)	
H201	0.1960	0.6349	0.4910	0.067*	
H202	0.0599	0.5802	0.5822	0.067*	
C21	0.2923 (4)	0.4523 (3)	0.6457 (2)	0.0502 (6)	
H211	0.3980	0.4667	0.6411	0.060*	
H212	0.3022	0.3805	0.6022	0.060*	
C22	0.3782 (3)	0.2403 (3)	0.8511 (3)	0.0480 (5)	
H221	0.3960	0.1769	0.7969	0.058*	
H222	0.4798	0.2588	0.8566	0.058*	
C23	0.3262 (3)	0.1631 (2)	0.9849 (2)	0.0423 (5)	
C24	0.3844 (4)	0.1675 (3)	1.0962 (3)	0.0563 (6)	
H241	0.4594	0.2183	1.0883	0.068*	
C25	0.3348 (4)	0.0959 (3)	1.2180 (3)	0.0658 (8)	
H251	0.3758	0.1010	1.2910	0.079*	
C26	0.2275 (4)	0.0170 (3)	1.2299 (3)	0.0629 (7)	
H261	0.1933	−0.0309	1.3131	0.075*	
C27	0.1682 (4)	0.0116 (3)	1.1200 (3)	0.0606 (7)	
H271	0.0944	−0.0426	1.1291	0.073*	
C28	0.2175 (3)	0.0838 (3)	0.9975 (2)	0.0496 (5)	
H281	0.1822	0.0763	0.9236	0.059*	
Cl1	0.19840 (7)	0.07672 (7)	0.62088 (5)	0.0459 (2)	
O1A	0.0878 (13)	0.0586 (10)	0.5438 (9)	0.071 (2)	0.66 (3)
O2A	0.2349 (11)	−0.0512 (9)	0.7360 (6)	0.0630 (17)	0.66 (3)
O3A	0.1333 (19)	0.2126 (12)	0.6492 (15)	0.095 (4)	0.66 (3)
O4A	0.3513 (9)	0.0646 (13)	0.5527 (11)	0.086 (3)	0.66 (3)
O1B	0.122 (3)	0.082 (3)	0.510 (2)	0.101 (7)	0.34 (3)
O2B	0.190 (4)	−0.047 (2)	0.724 (2)	0.105 (6)	0.34 (3)
O3B	0.099 (2)	0.2007 (17)	0.660 (2)	0.064 (4)	0.34 (3)
O4B	0.3506 (16)	0.098 (2)	0.585 (2)	0.080 (3)	0.34 (3)
Cl2	−0.23663 (8)	0.44332 (8)	0.79535 (8)	0.0570 (2)	
O5	−0.2245 (5)	0.5724 (5)	0.6812 (5)	0.1421 (15)	
O6	−0.3698 (4)	0.5028 (5)	0.8630 (4)	0.1273 (13)	
O7	−0.2735 (4)	0.3526 (4)	0.7389 (3)	0.0951 (9)	
O8	−0.0873 (3)	0.3889 (4)	0.8630 (3)	0.0991 (10)	
O9	0.4548 (3)	−0.2670 (3)	0.5678 (3)	0.0757 (7)	
H91	0.4625	−0.1874	0.5194	0.114*	
H92	0.5408	−0.3200	0.6072	0.114*	

### Atomic displacement parameters (Å^2^)

**Table:**

	*U*^11^	*U*^22^	*U*^33^	*U*^12^	*U*^13^	*U*^23^
N16	0.0336 (8)	0.0422 (9)	0.0420 (9)	−0.0174 (7)	0.0030 (7)	−0.0149 (7)
N19	0.0463 (11)	0.0441 (10)	0.0544 (11)	−0.0149 (8)	−0.0041 (9)	−0.0050 (8)
C17	0.0433 (11)	0.0430 (10)	0.0421 (10)	−0.0128 (9)	−0.0001 (8)	−0.0181 (9)
C18	0.0464 (12)	0.0436 (11)	0.0537 (12)	−0.0111 (9)	0.0049 (10)	−0.0162 (10)
C20	0.0661 (17)	0.0639 (15)	0.0410 (11)	−0.0303 (13)	−0.0058 (11)	−0.0096 (10)
C21	0.0606 (15)	0.0569 (14)	0.0429 (11)	−0.0285 (12)	0.0128 (10)	−0.0222 (10)
C22	0.0369 (10)	0.0437 (11)	0.0629 (14)	−0.0112 (9)	0.0065 (10)	−0.0200 (10)
C23	0.0362 (10)	0.0365 (9)	0.0527 (11)	−0.0076 (8)	−0.0031 (9)	−0.0154 (8)
C24	0.0560 (14)	0.0472 (12)	0.0656 (15)	−0.0145 (11)	−0.0176 (12)	−0.0147 (11)
C25	0.077 (2)	0.0540 (15)	0.0563 (15)	−0.0025 (14)	−0.0229 (14)	−0.0163 (12)
C26	0.0640 (17)	0.0517 (14)	0.0544 (14)	−0.0049 (12)	0.0031 (12)	−0.0081 (11)
C27	0.0546 (15)	0.0554 (14)	0.0694 (17)	−0.0229 (12)	0.0045 (13)	−0.0132 (13)
C28	0.0476 (12)	0.0539 (13)	0.0515 (12)	−0.0201 (10)	−0.0020 (10)	−0.0177 (10)
Cl1	0.0433 (3)	0.0519 (3)	0.0484 (3)	−0.0204 (2)	−0.0019 (2)	−0.0177 (2)
O1A	0.079 (4)	0.062 (3)	0.078 (3)	−0.032 (3)	−0.033 (3)	−0.010 (3)
O2A	0.073 (4)	0.065 (3)	0.044 (2)	−0.030 (2)	−0.007 (2)	0.0004 (17)
O3A	0.123 (8)	0.077 (4)	0.112 (6)	−0.038 (4)	−0.016 (5)	−0.050 (4)
O4A	0.074 (3)	0.085 (5)	0.088 (5)	−0.039 (3)	0.015 (3)	−0.005 (3)
O1B	0.088 (10)	0.134 (16)	0.104 (11)	−0.017 (9)	−0.025 (9)	−0.078 (10)
O2B	0.130 (15)	0.093 (10)	0.125 (12)	−0.088 (11)	0.070 (9)	−0.047 (8)
O3B	0.060 (6)	0.054 (5)	0.071 (6)	0.003 (5)	−0.005 (4)	−0.029 (5)
O4B	0.060 (5)	0.094 (7)	0.116 (9)	−0.047 (5)	0.029 (5)	−0.057 (7)
Cl2	0.0410 (3)	0.0587 (4)	0.0838 (5)	−0.0185 (3)	−0.0003 (3)	−0.0358 (3)
O5	0.122 (3)	0.127 (3)	0.160 (3)	−0.076 (2)	−0.022 (2)	0.018 (2)
O6	0.0690 (18)	0.192 (3)	0.159 (3)	−0.029 (2)	0.0164 (18)	−0.120 (3)
O7	0.111 (2)	0.112 (2)	0.1031 (19)	−0.0633 (19)	0.0164 (17)	−0.0613 (18)
O8	0.0527 (14)	0.133 (3)	0.103 (2)	−0.0300 (15)	−0.0070 (13)	−0.0256 (19)
O9	0.0635 (14)	0.0607 (12)	0.1013 (17)	−0.0305 (11)	0.0107 (12)	−0.0167 (12)

### Geometric parameters (Å, °)

**Table:**

N16—C21	1.491 (3)	C24—C25	1.377 (4)
N16—C17	1.499 (3)	C24—H241	0.9313
N16—C22	1.519 (3)	C25—C26	1.379 (5)
N16—H161	0.8954	C25—H251	0.9269
N19—C18	1.486 (3)	C26—C27	1.374 (5)
N19—C20	1.498 (4)	C26—H261	0.9388
N19—H191	0.8898	C27—C28	1.384 (4)
N19—H192	0.8904	C27—H271	0.9459
C17—C18	1.510 (3)	C28—H281	0.9242
C17—H171	0.9685	Cl1—O2B	1.382 (12)
C17—H172	0.9713	Cl1—O4B	1.398 (11)
C18—H181	0.9500	Cl1—O1B	1.409 (12)
C18—H182	0.9681	Cl1—O3A	1.416 (8)
C20—C21	1.503 (4)	Cl1—O2A	1.427 (5)
C20—H201	0.9532	Cl1—O3B	1.430 (10)
C20—H202	0.9768	Cl1—O1A	1.430 (6)
C21—H211	0.9671	Cl1—O4A	1.437 (7)
C21—H212	0.9645	Cl2—O6	1.374 (3)
C22—C23	1.500 (3)	Cl2—O7	1.386 (3)
C22—H221	0.9721	Cl2—O8	1.405 (3)
C22—H222	0.9695	Cl2—O5	1.484 (4)
C23—C24	1.380 (3)	O9—H91	0.8131
C23—C28	1.390 (3)	O9—H92	0.8189
			
C21—N16—C17	109.70 (17)	N16—C22—H222	108.0
C21—N16—C22	110.81 (18)	H221—C22—H222	108.4
C17—N16—C22	111.48 (17)	C24—C23—C28	118.9 (2)
C21—N16—H161	107.9	C24—C23—C22	121.7 (2)
C17—N16—H161	109.9	C28—C23—C22	119.3 (2)
C22—N16—H161	107.0	C25—C24—C23	120.8 (3)
C18—N19—C20	110.79 (19)	C25—C24—H241	120.0
C18—N19—H191	110.7	C23—C24—H241	119.2
C20—N19—H191	110.0	C24—C25—C26	120.0 (3)
C18—N19—H192	110.1	C24—C25—H251	118.8
C20—N19—H192	109.1	C26—C25—H251	121.1
H191—N19—H192	106.1	C27—C26—C25	119.9 (3)
N16—C17—C18	111.57 (19)	C27—C26—H261	120.3
N16—C17—H171	108.2	C25—C26—H261	119.7
C18—C17—H171	109.2	C26—C27—C28	120.1 (3)
N16—C17—H172	108.2	C26—C27—H271	119.3
C18—C17—H172	110.4	C28—C27—H271	120.5
H171—C17—H172	109.2	C27—C28—C23	120.2 (2)
N19—C18—C17	111.1 (2)	C27—C28—H281	120.4
N19—C18—H181	107.3	C23—C28—H281	119.4
C17—C18—H181	111.0	O2B—Cl1—O4B	119.3 (15)
N19—C18—H182	108.7	O2B—Cl1—O1B	109.0 (11)
C17—C18—H182	109.9	O4B—Cl1—O1B	109.0 (12)
H181—C18—H182	108.7	O3A—Cl1—O2A	112.5 (8)
N19—C20—C21	110.0 (2)	O2B—Cl1—O3B	104.2 (14)
N19—C20—H201	110.1	O4B—Cl1—O3B	107.6 (11)
C21—C20—H201	108.4	O1B—Cl1—O3B	107.0 (13)
N19—C20—H202	110.0	O3A—Cl1—O1A	111.9 (7)
C21—C20—H202	108.6	O2A—Cl1—O1A	107.1 (4)
H201—C20—H202	109.7	O3A—Cl1—O4A	112.7 (6)
N16—C21—C20	111.0 (2)	O2A—Cl1—O4A	104.5 (5)
N16—C21—H211	109.0	O1A—Cl1—O4A	107.8 (6)
C20—C21—H211	110.0	O6—Cl2—O7	109.7 (2)
N16—C21—H212	109.1	O6—Cl2—O8	114.0 (2)
C20—C21—H212	110.5	O7—Cl2—O8	118.4 (2)
H211—C21—H212	107.1	O6—Cl2—O5	104.5 (3)
C23—C22—N16	111.90 (18)	O7—Cl2—O5	103.1 (2)
C23—C22—H221	109.6	O8—Cl2—O5	105.4 (2)
N16—C22—H221	108.8	H91—O9—H92	111.3
C23—C22—H222	110.1		

### Hydrogen-bond geometry (Å, °)

**Table:**

*D*—H···*A*	*D*—H	H···*A*	*D*···*A*	*D*—H···*A*
O9—H91···O4A^i^	0.81	2.27	2.942 (11)	141
O9—H92···O5^ii^	0.82	2.06	2.869 (6)	170
N16—H161···O8	0.90	2.15	2.964 (3)	151
N19—H191···O9^iii^	0.89	1.92	2.750 (4)	155
N19—H192···O1A^iv^	0.89	2.08	2.907 (10)	154
C17—H172···O6^v^	0.97	2.48	3.446 (5)	172
C20—H201···O7^iv^	0.95	2.49	3.406 (4)	160
C21—H212···O3A	0.96	2.48	3.130 (15)	125

Symmetry codes: (i) −*x*+1, −*y*, −*z*+1; (ii) *x*+1, *y*−1, *z*; (iii) *x*, *y*+1, *z*; (iv) −*x*, −*y*+1, −*z*+1; (v) *x*+1, *y*, *z*.
